# Selectivity profiling of BCRP versus P-gp inhibition: from automated collection of polypharmacology data to multi-label learning

**DOI:** 10.1186/s13321-016-0121-y

**Published:** 2016-02-04

**Authors:** Floriane Montanari, Barbara Zdrazil, Daniela Digles, Gerhard F. Ecker

**Affiliations:** Pharmacoinformatics Research Group, Department of Pharmaceutical Chemistry, University of Vienna, Althanstraße 14, 1090 Vienna, Austria

**Keywords:** BCRP, P-glycoprotein, Open Data, Multi-label classification, Binary relevance, Classifiers chain, Selective inhibition, Polyspecific inhibition, KNIME, Open PHACTS

## Abstract

**Background:**

The human ATP binding cassette transporters Breast Cancer Resistance Protein (BCRP) and Multidrug Resistance Protein 1 (P-gp) are co-expressed in many tissues and barriers, especially at the blood–brain barrier and at the hepatocyte canalicular membrane. Understanding their interplay in affecting the pharmacokinetics of drugs is of prime interest. *In silico* tools to predict inhibition and substrate profiles towards BCRP and P-gp might serve as early filters in the drug discovery and development process. However, to build such models, pharmacological data must be collected for both targets, which is a tedious task, often involving manual and poorly reproducible steps.

**Results:**

Compounds with inhibitory activity measured against BCRP and/or P-gp were retrieved by combining Open Data and manually curated data from literature using a KNIME workflow. After determination of compound overlap, machine learning approaches were used to establish multi-label classification models for BCRP/P-gp. Different ways of addressing multi-label problems are explored and compared: label-powerset, binary relevance and classifiers chain. Label-powerset revealed important molecular features for selective or polyspecific inhibitory activity. In our dataset, only two descriptors (the numbers of hydrophobic and aromatic atoms) were sufficient to separate selective BCRP inhibitors from selective P-gp inhibitors. Also, dual inhibitors share properties with both groups of selective inhibitors. Binary relevance and classifiers chain allow improving the predictivity of the models.

**Conclusions:**

The KNIME workflow proved a useful tool to merge data from diverse sources. It could be used for building multi-label datasets of any set of pharmacological targets for which there is data available either in the open domain or in-house. By applying various multi-label learning algorithms, important molecular features driving transporter selectivity could be retrieved. Finally, using the dataset with missing annotations, predictive models can be derived in cases where no accurate dense dataset is available (not enough data overlap or no well balanced class distribution).

**Graphical abstract:**
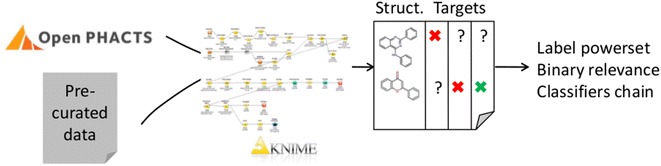
.

**Electronic supplementary material:**

The online version of this article (doi:10.1186/s13321-016-0121-y) contains supplementary material, which is available to authorized users.

## Background

Typically, collecting a dataset of pharmacological annotations between compounds and a given target is a tedious task. Researchers must browse diverse Open Databases like ChEMBL [1] or PubChem [2], manually extract data from published papers and then take decisions on how to merge and combine the different data sources. When more than one target is involved, then the work becomes even harder.

Here, we present an automatic workflow that combines pre-curated datasets (either in-house or literature-retrieved and manually curated) with data in the Open PHACTS Discovery Platform [3] on different targets. Data merging can be customized and priority can be given to the pre-curated data. There is no limit on the number of targets and sources from which data can be retrieved.

The workflow was applied to retrieve a two-target dataset for BCRP and P-gp inhibition. P-gp and BCRP are two members of the superfamily of ABC-transporters. These transmembrane proteins hydrolyze ATP to fuel the export of their substrates out of the cells (for a review, see [4]). BCRP and P-gp are co-expressed at various barriers throughout the human body, especially at the blood–brain barrier and liver canalicular membrane. Both are known to play a role in multiple drug resistance (MDR), a syndrome by which cancer cells become resistant to a broad panel of cytotoxic compounds [5]. In addition, they play a major role in causing clinically relevant drug–drug interactions (DDIs) by altering the pharmacokinetic profiles of many co-administered drugs [6]. At the blood–brain barrier, the transporters decrease the bioavailability of their substrates in the brain, which can be a desired or unwanted property depending on the drug. In the hepatocytes, they help eliminating xenobiotics. Thus, inhibition of the two transporters could lead to increased xenobiotics concentration in the liver, which results in hepatotoxicity. For these reasons, models predicting the inhibition of the two transporters by small molecules are helpful to detect potential drug–drug interaction perpetrators. Both transporters have been extensively studied independently, and several machine learning models to predict BCRP or P-gp inhibition have been published and reviewed [7–9]. However, no attempt has been made so far to build a model for both transporters simultaneously in a multi-label approach. Such an approach appears suitable due to the diverse and partly overlapping inhibitor profiles of those promiscuous efflux pumps. Thus, by using the inherent information of these common inhibitor profiles for multi-label classification modeling, one could shed light on the chemical preferences of each transporter and understand what renders a molecule a dual or a selective inhibitor of BCRP or P-gp.

Using the aforementioned data extraction workflow, a dataset with pharmacological annotations for BCRP and/or P-gp inhibition was obtained. The majority of compounds have a known annotation only for one of the two targets, while a small amount contains activity data for both. Building models with this type of data is referred to as “multi-label learning” [10]. Different ways to tackle such a problem have been reviewed in the field of machine learning [11, 12]. “Binary relevance” decomposes multi-label classification into multiple independent binary classifications (one per label), and joins the output predictions a posteriori. Another way to tackle the problem is “label-powerset”, which transforms all the occurrences of label combinations in the data into individual classes to obtain a multi-class problem. However, this method may get too complex for problems with many labels, since label combinations (hence the number of classes in the transformed multi-class problem) increase exponentially with the number of labels. In 2009, Read and colleagues [13] introduced “classifiers chain”, a modification of binary relevance that would take into account interactions between labels. The concept is still to build one model per label, but this time sequentially: first, one label is randomly chosen to build the first model. This model is used to make predictions on the whole dataset. These predictions are then added to the descriptor matrix to train the next model on another randomly picked label. The process goes on until all labels have been used to build a model.

In this work, we apply the three methods (label-powerset, binary relevance and classifiers chain) to a large BCRP/P-gp inhibition dataset derived from various sources. First, the models built using label-powerset allow finding physicochemical molecular descriptors that best distinguish selective from dual inhibitors. Then, improved predictability is gained by using either classifiers chain or binary relevance transformations. These methods seem most useful when little annotation overlap is present in the data and/or many targets are to be predicted.

## Results and discussion

### Data retrieval and analysis

**Fig. 1:**
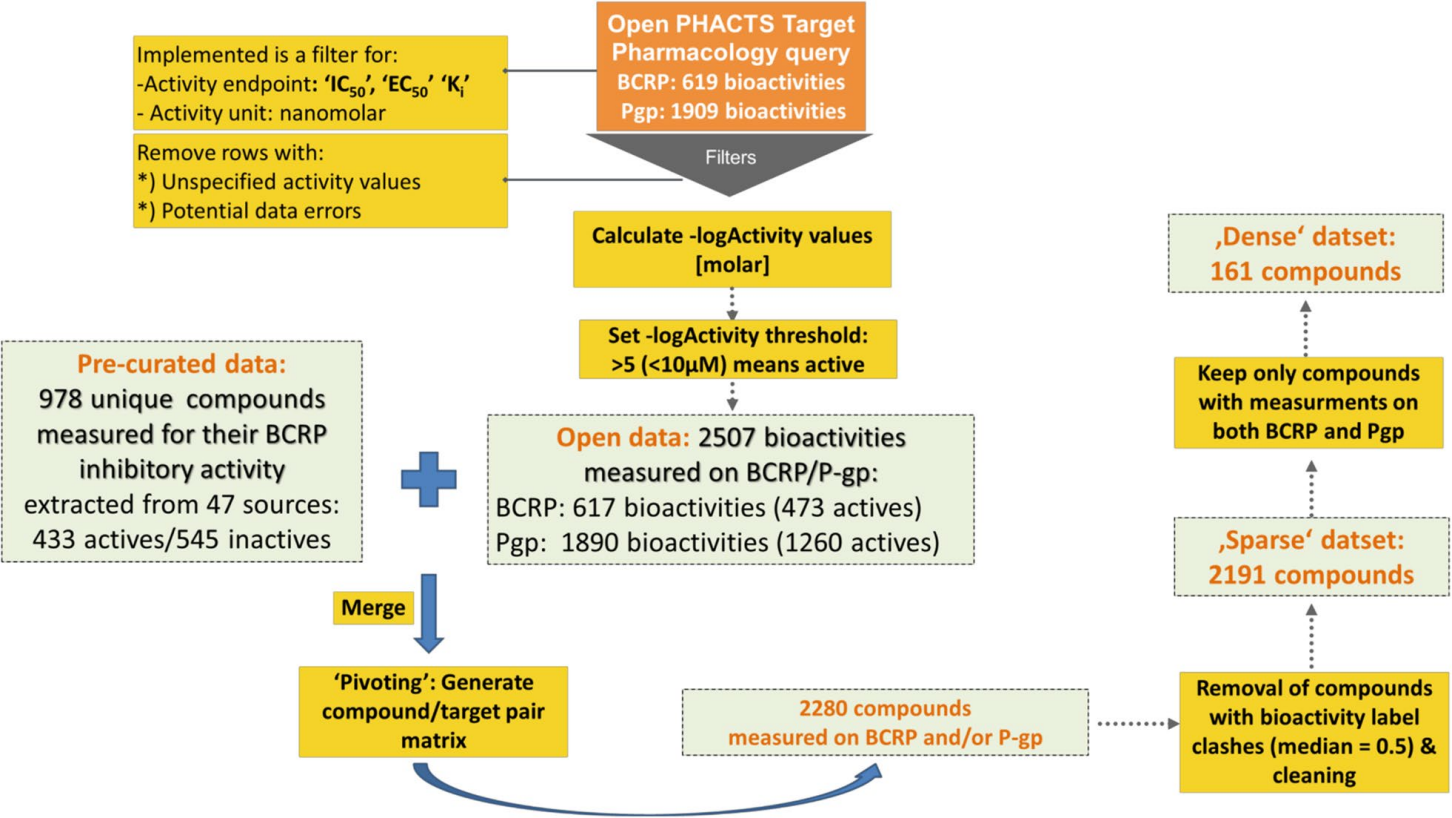
Depiction of the data collection workflow

In this paper, we are presenting a semi-automatic, fully flexible KNIME workflow [14] for data retrieval, merging, pre-processing, filtering, depiction and output. The most important steps are illustrated in Fig. 1. The workflow showed special usefulness for collating data from various sources, including various protein targets as well as datasets generated under various conditions. In this use case, P-gp and BCRP data are retrieved from the Open PHACTS Discovery Platform [3] as well as from a pre-curated literature dataset. From the Open PHACTS Discovery Platform, 617 compounds annotated for BCRP inhibition (473 inhibitors, 144 non-inhibitors) as well as 1890 compounds annotated for P-gp inhibition (1260 inhibitors, 630 non-inhibitors) were initially retrieved. The pre-curated literature dataset contains 978 molecules annotated for BCRP inhibition (433 inhibitors, 545 non-inhibitors) [15]. Taken together, the data collation started with 3485 measurements for BCRP and P-gp. By creating an overlap matrix via InChIKeys, 2280 unique molecules were retained. Next, removal of entries with ambiguous activity labels and data cleaning led to the sparse dataset of 2191 compounds: 1104 have an activity reported for BCRP (533 inhibitors, 571 non-inhibitors) and 1248 have an activity reported for P-gp (847 inhibitors, 401 non-inhibitors).

The dense dataset contains only those compounds of the sparse dataset where there are activity labels available for both BCRP and P-gp (161 compounds). A binary pharmacological heat map representation—retrieved directly from our KNIME workflow—of the dense dataset for inhibitory activity against BCRP/P-gp is available as Additional file 1: Figure SI-1. Table 1 shows the exact amount of compounds in each of the four classes (see also “Methods” section for an exact definition of the four classes). Overall, the classes show a well balanced distribution. The class with the lowest number of representatives (27) is the collection of compounds which are non-inhibitors towards both transporters.

**Table 1:** Number of compounds belonging to each class of the BCRP/P-gp multi-class dataset

Class	Number of compounds	Inhibitors of P-gp	Inhibitors of BCRP	Description
0	27	No	No	Non-inhibitors
1	48	Yes	No	P-gp-selective inhibitors
2	39	No	Yes	BCRP-selective inhibitors
3	47	yes	Yes	Dual inhibitors

**Fig. 2:**
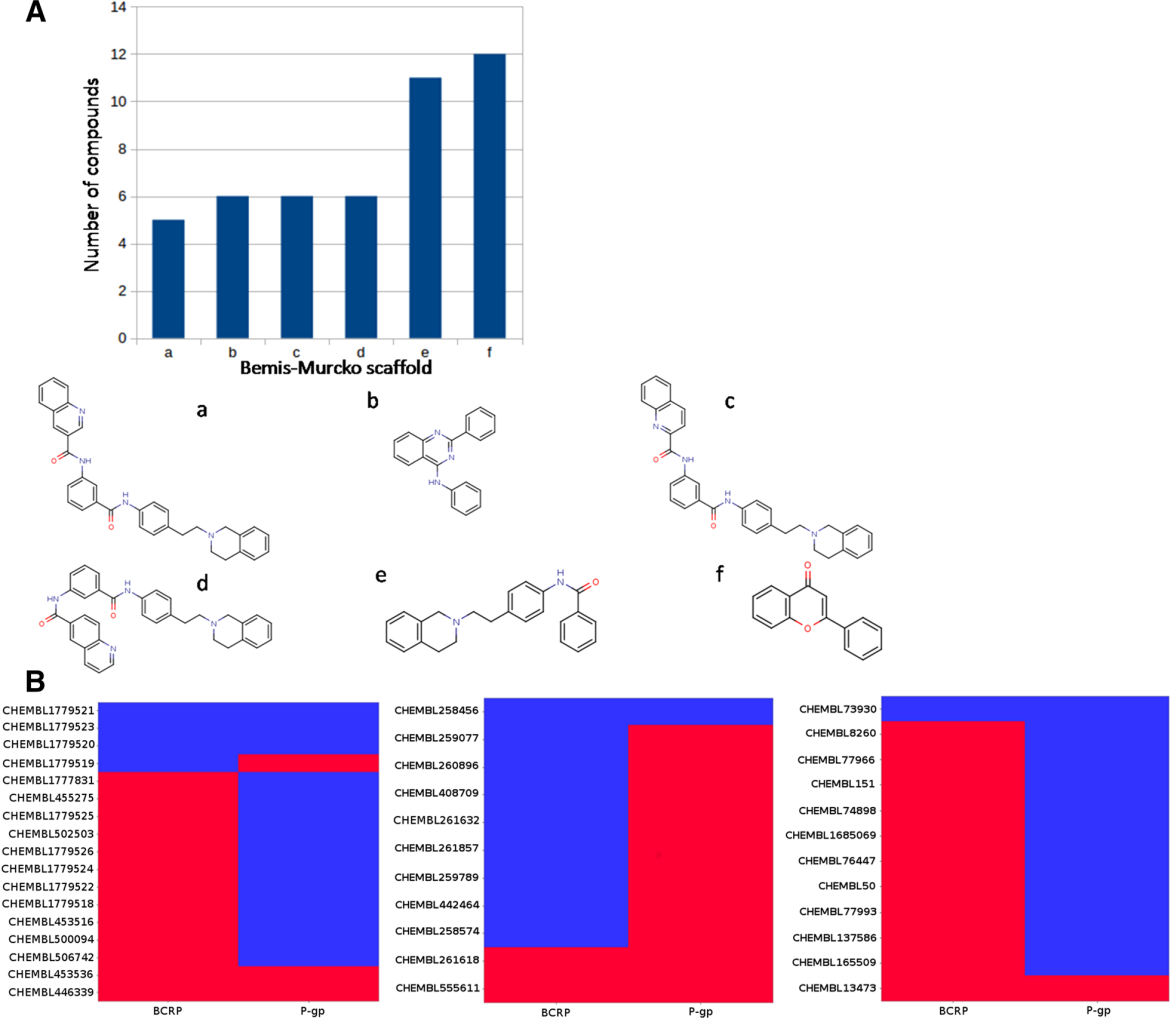
Analysis of the scaffolds present in at least five compounds of the dense dataset. **A** On *top left* distribution of compounds sharing the scaffolds. *Down* depiction of the six scaffolds (**a**–**f**). **B** Binary heat map representations of inhibitory activities for BCRP and P-gp of the compounds sharing scaffolds **a**, **c** and **d** (left heat map), scaffold **e** (middle heat map) or **f** (right heat map): *red bars* inhibitors; *blue bars* non-inhibitors; abscissae: targets; ordinates: compounds annotated with ChEMBL compound IDs

Analyzing the 161-compound (dense) dataset by extracting their Bemis–Murcko frameworks [16] resulted in 97 unique scaffolds, which corresponds to an average of 1.6 molecules per distinct scaffold. In reality, 75 scaffolds have only one representative compound, and only six scaffolds have at least five representative compounds (Fig. 2).

A closer inspection of scaffolds a, c and d reveals that the single structural difference is the position of the amide substituent on the quinoline ring system. Therefore, scaffold clusters a, c, and d were merged into one cluster, now containing 17 compounds. As seen from the pharmacological heat map representations in Fig. 2B, there is a certain trend for preferred activity against BCRP within this cluster. In scaffolds e and f, the binding preference is even more pronounced (see Fig. 2B): cluster e seems to be rather P-gp selective, while cluster f shows a rather BCRP selective pharmacological profile. Exceptions to these homogeneous pharmacological profiles towards BCRP/P-gp in clusters e and f could give clues about structure–activity relationships and selectivity switches. In some cases, however, the activity was on the border of the 10 µM cutoff set for separating active from inactive (12 µM for compound ChEMBL73930 and 19 µM for compound ChEMBL258456), and could also point to incoherencies between different assay setups, for example. Apart from the enriched scaffold clusters, which comprise 46 compounds in total, the dense dataset can be considered as structurally diverse with respect to scaffold variety.

**Fig. 3:**
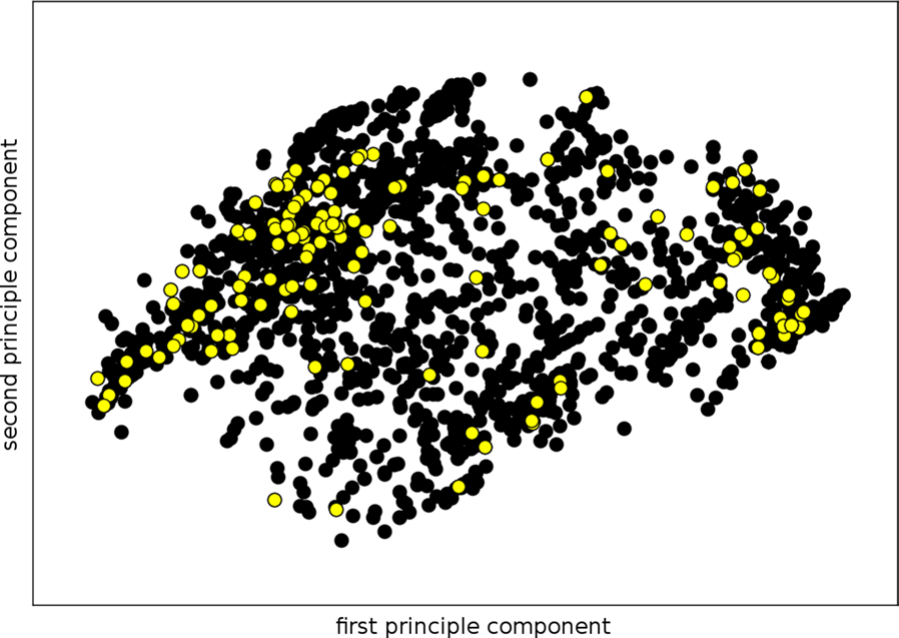
Projection of the dense dataset (*yellow dots*) over the PCA transformations obtained for the sparse dataset (*black dots*) using MACCS fingerprints

The sparse dataset contains 2191 compounds, with 997 unique Bemis–Murcko scaffolds, which corresponds to an average of 2.2 molecules per distinct scaffold. On a closer look, over 650 scaffolds have only one representative compound, 91 scaffolds have at least five representative compounds and only 13 scaffolds have more than 20 representative compounds (these highly represented scaffolds are plotted in Additional file 1: Figure SI-2 including an overview of the class repartition among the scaffolds). This, again, underpins the datasets structural diversity. To compare the chemical space of the two datasets under study, the molecules were encoded into MACCS fingerprints and a principle components analysis (PCA) was performed on the sparse dataset. The dense dataset was projected using the transformation obtained with the sparse dataset, and the first two principal components were used to depict the data (Fig. 3). The result shows good overlap of the two projections, giving us the idea that the chemical spaces of the two datasets are not fundamentally different. The same approach was additionally performed with ECFP-like fingerprints and the figure is available as Additional file 1: Figure SI-3.

### Four-class classification: categorizing at once

The dense dataset retrieved in this study is not large (161 unique compounds), but, as opposed to other multi-label classification approaches that consider an absence of biological annotation as a negative [17], we here focus on a dataset for which each inhibition and absence of inhibition has been measured in an in vitro assay. This is a crucial point, especially for targets such as the promiscuous ABC-transporters: one cannot simply assume that a lack of data means an absence of activity for that compound on that target. The presence of annotated data for both BCRP inhibition and P-gp inhibition was encoded into four classes using the label-powerset method (see “Methods” section). Having only two labels (BCRP and P-gp) is an advantage for this technique as the number of classes derived from such transformation is 2^|L|^ (where |L| is the number of labels in the initial dataset).

In a first instance, we were interested in predicting all of the four classes at once. The multi-class classifier thus learns to distinguish inhibitors of BCRP from inhibitors of both BCRP and P-gp and so on. It is a complex task that was tackled with different learning algorithms implemented in Weka. The first choice was a multilayer perceptron with four output nodes (one for each class), as no transformation is needed to treat multi-class problems. However, results were not promising and the computational time needed was higher than those for algorithms with similar accuracy (Table 2).

**Table 2:** 10-fold cross-validation results of selected classifiers built on the 4-class classification dataset

Algorithm	Descriptor set	Accuracy (%)	Kappa	AUC
MultilayerPerceptron, training time = 600	MOE2D	55.3	0.39	0.81
MultilayerPerceptron, training time = 600	MACCS	59.0	0.44	0.79
K nearest neighbours, k = 5	MOE2D	60.2	0.46	0.80
K nearest neighbours, k = 5	MACCS	57.1	0.41	0.78
K nearest neighbours, k = 5	ECFP	54.7	0.40	0.80
RotationForest, iterations = 50	MOE2D	60.9	0.47	0.85
RotationForest, iterations = 50	MACCS	62.7	0.50	0.84
SVM with polynomial kernel	MOE2D	57.1	0.42	0.78
SVM with polynomial kernel	MACCS	62.7	0.49	0.80
*SVM with polynomial kernel*	*ECFP*	*70.2*	*0.60*	*0.84*
RandomForest, 200 trees	MOE2D	60.9	0.47	0.85
RandomForest, 200 trees	MACCS	60.9	0.47	0.86
RandomForest, 200 trees	ECFP	67.1	0.55	0.88

When no results are shown for the descriptor set ECFP, it is because the computational time and/or memory needed were too large. In italic letters, the model that gives the best cross-validation results

As can be seen, the overall classification results are mediocre. This is probably due to the small amount of data available for training the classifier and the harsh way of computing the accuracy (the classifier has to predict properly not only two but four distinct classes). According to the confusion matrices (data not shown), it seems that the classifiers essentially fail to properly predict class 3, often mistaken for a class 1 or class 2. The best model was obtained for the ECFP descriptor set, in the polynomial support vector machine setting.

### Three-class classification: distinguishing between different types of transport inhibitors

Three-class classification would allow us to understand what differentiates the selective inhibitors of classes 1 and 2 from the polyspecific inhibitors of class 3. Thus, the aim is not to reach a powerful model, but rather to interpret the models and maybe get a clearer idea as to what makes an inhibitor selective or not. We therefore focused on a subset of 71 interpretable MOE descriptors and learning algorithms that lead to interpretable models. Only data labeled as classes 1, 2 or 3 was used for training the models.

Important features can be retrieved easily from the J48 trees and from the Jrip rules. For the first three models (see Table 3), descriptors related to hydrophobicity (SlogP and a_hyd), the number of aromatic atoms (a_aro), the Van der Waals surface area of H bond acceptors (vsa_acc) and Balaban’s connectivity topological index (BalabanJ) were identified as important descriptors for distinguishing the three classes. For the bagging models, the feature importances for each of the 50 trees were averaged (see “Methods”), and the most important descriptors extracted were similar to the ones from the J48 trees: SlogP, a_hyd, a_aro, as well as the number of donor and acceptor atoms (a_donacc), and the Wiener path (weinerPath; sum of the lengths of the shortest paths between all pairs of heavy atoms). Thus, in total we focused on seven different descriptors having been identified as important ones. The distribution of these important features was explored in order to understand in which direction they influence selectivity.

**Table 3:** Average over ten ten-fold cross-validation runs for given models trained on the 3-class classification subset

Algorithm	Embedded feature selection	Accuracy (%)	Kappa	AUC
J48 tree	Yes	61.6	0.42	0.73
J48 tree, min. 5 instances/leaf	Yes	59.1	0.38	0.73
Jrip rules	Yes	57.8	0.36	0.70
Bagging of J48	No	65.0	0.47	0.82
Bagging of J48	Yes	62.6	0.44	0.80

**Fig. 4:**
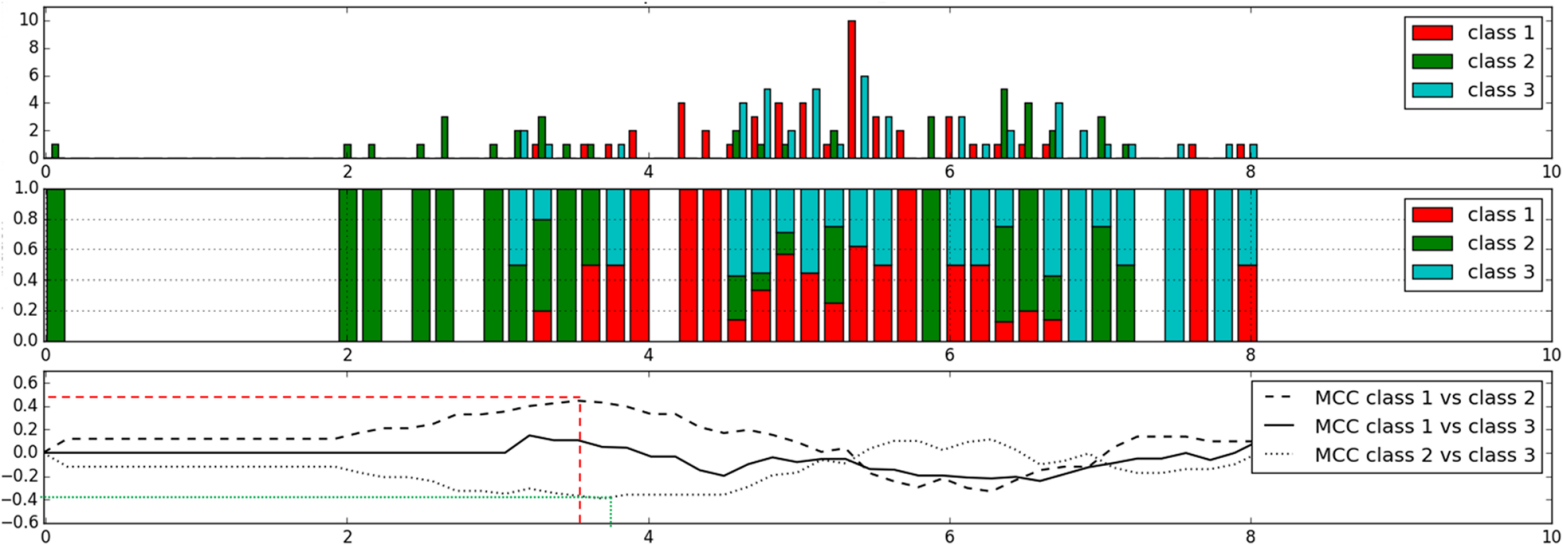
Distribution of SlogP among the three kinds of inhibitors. Inhibitors of P-gp only: *red bars* (class 1); inhibitors of BCRP only: *green bars* (class 2); inhibitors of both P-gp and BCRP: *blue bars* (class 3). *Top panel* bar plot of the counts per binned value of SlogP. *Middle panel* proportions of each class in each bin, by putting each bin count to 100 %. *Lower panel* Matthews Correlation Coefficient (MCC) that would be obtained by splitting the data at each SlogP value. MCC values that peak above or below 0 show ideal thresholds to separate the data between classes. The *colored dotted lines* corresponds to the peaks of MCC and the corresponding SlogP values (between 3 and 4) for separating class 1 from 2 (*red dotted lines*) and class 2 from 3 (*green dotted lines*)

As exemplified in Fig. 4, showing the distribution of SlogP among classes 1, 2 and 3, inhibitors of BCRP only (green bars) tend to take either low or high values of SlogP, while inhibitors of P-gp only (red bars) tend to take medium values of SlogP. The dual inhibitors (blue bars) overlap with the class 1 inhibitors in the medium values of SlogP and the with class 2 inhibitors in the higher values of SlogP. The three Matthews Correlation Coefficient curves (MCC, lower panel) show that separating class 1 (P-gp-selective inhibitors) from class 3 (dual inhibitors) is not possible using SlogP (rather flat profile of the continuous line). On the contrary, separating class 1 from class 2 is feasible with a threshold around SlogP = 3.5 (peak of the MCC discontinuous line at around 0.4). Finally, the best threshold to separate BCRP inhibitors from dual inhibitors is at SlogP = 3.5 (peak of the MCC dotted line at around −0.4). Of course, these thresholds are based only on one descriptor and would not produce an optimal separation.

Identical plots were generated for the other descriptors retrieved by the 3-class classification model as critical for the separation of the three types of inhibitors and are available as Additional file 1: Figures SI-4 to SI-9. In all cases, the separation of the dual inhibitors from one of the two classes of selective inhibitors is not trivial. These findings go into the same direction as what was found for the 4-class classification models: predicting class 3 (the dual inhibitors) is a difficult task.

**Fig. 5:**
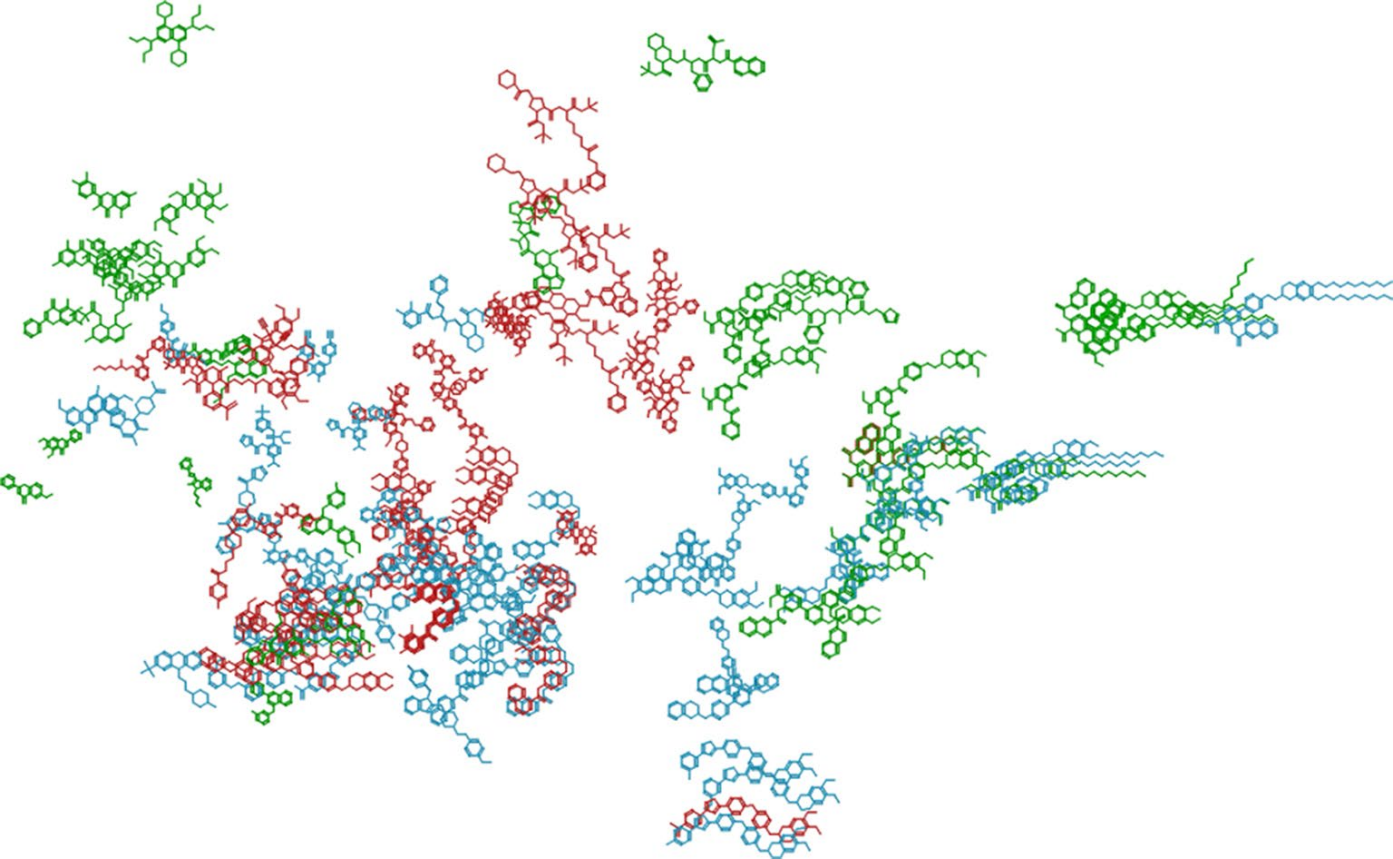
View of the 3D embedding proposed by CheS-Mapper using the descriptors SlogP, a_hyd, a_aro, vsa_acc, BalabanJ, a_donacc, weinerPath. In *red* P-gp-selective inhibitors (class 1), in *green* BCRP-selective inhibitors (class 2). In *blue* dual inhibitors (class 3)

To further validate the usefulness of the seven descriptors selected by the models altogether, we performed a three-dimensional embedding of the dense dataset (restricted to classes 1, 2 and 3) using ChesMapper [18] and the seven descriptors. The quality of the obtained embedding was very high (r^2^ = 0.97), which means that compounds with similar descriptor values would appear close together in space (Fig. 5) and that the seven descriptors are meaningful in the context of our dataset. In addition, a separation of the three classes can be seen on the embedding (especially classes 1 and 2 in green and red, Fig. 5), which confirms the selected descriptors as helpful to distinguish the three types of inhibitors.

Very recently, Egido and colleagues studied the relationship between the hydrophobicity, polarizability and charge with the capacity for a compound to be transported or to inhibit BCRP and/or P-gp by applying a semi-quantitative scoring scheme [19]. Using a calibrating set of 30 drugs, the ATPase activity of the two transporters was measured as a function of the concentration of the drugs. Dual inhibitors (only three compounds out of the 30 drugs) were hydrophilic, highly amphiphilic and non-charged. Our dataset contains 47 dual inhibitors, which are indeed non-charged. However, our set of dual inhibitors has, on average, a sum of atomic polarizabilities of 78.2 (descriptor “apol” in MOE), to compare with 76.9 for the other compounds (non-inhibitors and selective inhibitors). Also in case of the number of hydrophobic atoms (descriptor “a_hyd” in MOE), there is no significant difference between dual inhibitors (27.5) and other compounds (26.2). Thus, for our dataset the criteria mentioned above do not allow to separate dual inhibitors from other compounds.

### Two-class classification: exploring selectivity

**Fig. 6:**
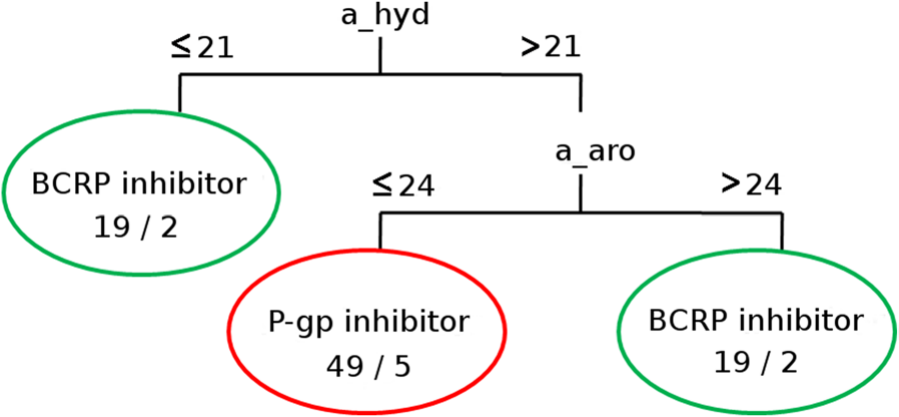
Tree depiction of the JRip model to separate P-gp-selective inhibitors (*red leaf*) from BCRP-selective inhibitors (*green leaves*). *a_hyd* number of hydrophobic atoms, *a_aro* number of aromatic atoms. The *numbers in the leaves* correspond to the number of compounds in the training set that ended up in that leaf (*left number*) and the number of compounds in the training set that were mispredicted in that leaf (*right number*)

Finally, we focused on the two sets of selective inhibitors: those that inhibit exclusively P-gp (class 1) and those that inhibit exclusively BCRP (class 2). A classification model using the restrained set of 71 MOE descriptors was built by training a simple set of rules (Jrip algorithm in Weka) with embedded attribute selection. The model obtained 82.1 % of accuracy, a kappa of 0.64 and an AUC of 0.81 (values are given as mean of ten ten-fold cross-validation runs). Interestingly, the model is very simple and is based only on two descriptors: the number of hydrophobic atoms (a_hyd) and the number of aromatic atoms (a_aro) (Fig. 6). Those two descriptors have also been identified as important ones in the previous three-class classification models. The rule set can be written as follows:

- if the number of hydrophobic atoms is less or equal to 21: then the compound would be selective for BCRP (class 2)
- else, if the number of aromatic atoms is superior or equal to 24: then the compound would be selective for BCRP (class 2)
- else: the compound is selective for P-gp (class 1)

Interestingly, in a very recent paper, Xu and colleagues demonstrated that π–π stacking plays a role in increasing the efficiency of binding of BCRP modulators [20]. This finding is in line with the higher number of aromatic atoms in selective BCRP inhibitors according to our machine learning model (second rule).

This model is not aimed at predicting new compounds, since one would first need to be sure that they are either inhibitors of P-gp or BCRP. Instead, it allows understanding, with the dataset at hand, what distinguishes the two kinds of selective inhibitors.

Applying these rules to the training set leads to 9 misclassifications. These rules encode the fact that, in the restricted dataset of selective compounds, the inhibitors of BCRP have more aromatic atoms, while the inhibitors of P-gp contain more hydrophobic atoms. Six out of nine compounds that were misclassified by the model were violating at least one Lipinski rule (regarding the molecular weight and lipophilicity) [21]. All misclassified structures are reported in Additional file 1: Figure SI-10. One could argue that the number of aromatic atoms and the number of hydrophobic atoms may be correlated in our data. This is not the case as can be seen on the plot of both descriptors in Additional file 1: Figure SI-11. During the dense dataset analysis, we discovered three scaffolds, together containing around 40 compounds, which showed some selectivity for P-gp or BCRP. Thus, those compounds are labeled as class 1 or 2 and are part of the training set used to build the two-class model. Therefore, the high cross-validation results and the simplicity of the obtained model could be due to the lack of chemical diversity in the training set, and reduce the applicability of the rules for any selective inhibitors of the two transporters of interest. Nevertheless, the model describes well the data available so far in the literature and in public databases.

### Chain of classifiers and binary relevance: exploiting all the data

The problem with the label-powerset approach described in the previous section is the need for data overlap between the labels. Here, the dataset got reduced to 161 compounds only. Adding just one other label, BSEP inhibition for example (another ABC-transporter expressed in the liver), would lead to 8 different classes to predict and much less than 100 compounds to train on.

In this section, we compare two methods that can handle missing annotations in the labels: binary relevance and classifiers chains. Binary relevance assumes that the labels are independent and would not bring any information to one another. Classifiers chains assume that having information for one label may help building the model for the next label. If one employs the same classifiers for both techniques, then the difference should come from the influence of using the information from one label to another in the case of the classifiers chain. The binary relevance performance can be considered as the baseline in this case.

When taking all data available with inhibition data in BCRP or P-gp, the dataset contains 2191 compounds, where most of the label annotations are missing for one of the transporters (sparse dataset).

Models were built on the sparse dataset either using the binary relevance protocol, or the classifiers chain protocol, keeping the same base classifiers inside (Logistic regression, RandomForest or SVM). Results are shown in Table 4. Performance is measured by ten-fold cross-validation in the sparse dataset.

**Table 4:** Comparison of results for binary relevance (baseline) and classifiers chain, by ten-fold cross-validation

Algorithms	Macro-accuracy	Macro-MCC	Macro-AUC
Binary relevance, Logistic Regression	0.812	0.594	0.793
Classifiers chain, Logisitic Regression	0.812	0.594	0.793
Binary relevance, RandomForest	0.835	0.641	0.808
*Classifiers chain, RandomForest*	*0.836*	*0.643*	*0.809*
Binary relevance, SVM	0.766	0.504	0.749
Classifiers chain, SVM	0.767	0.504	0.750

In italic letters, the model that gives the best results

Results show higher accuracy for all models when compared with the multi-class classification (Table 2), although one should keep in mind that the way accuracy is computed is different in both approaches. In the multi-class classification, accuracy is computed by summing the correct predictions for all classes, and dividing it by the total number of predictions. In the classifiers chain or binary relevance cases, this computation is not possible (due to the fact that many compounds lack annotation for one label). Instead, the macro-accuracy is reported, which corresponds to an averaged accuracy of each individual model built (see “Methods” for more details).There is no significant difference between models built with binary relevance (i.e. independent models for each label whose output is then shown as a list of predictions) and with the chain of classifiers. This indicates that for such a small amount of labels, we are not gaining enough information as for impacting the second model. In our concrete case here, it seems that label-powerset was initially a good choice since the dataset only has two labels and a good balance between class repartitions. To make use of all the data, binary relevance is a simple method that performs well. We suppose that, in the case of a dataset with many labels, the classifiers chain would actually show better performance than the naive binary relevance protocol.

## Summary and conclusions

Polypharmacology profiling requires carefully collated, large datasets. In this work, we present a workflow to automatically retrieve and assemble polypharmacology data using the Open PHACTS Discovery Platform in conjunction with in-house or literature-retrieved pre-curated data. This workflow allows automatizing the otherwise tedious task of manually querying databases, downloading structures and activity points, comparing data, establishing thresholds, etc. This task is the starting point of any ligand-based approach to tackle one or more targets. The fact that it allows combining pre-curated data may render it even more interesting for pharmaceutical companies (having their own in-house data) or research groups having already established datasets. Adding more targets to the query does not complicate the workflow; the only limit is the data availability.

In our study, we focused on the efflux pumps BCRP and P-gp, which have been recognized to influence the pharmacokinetic profile of co-administered drugs, thus causing drug–drug interactions and unwanted side-effects. Due to their promiscuous nature regarding their compound profiles, and the significant overlap in substrate and inhibitor profiles of BCRP/P-gp, inter-protein similarities in ligand recognition appear quite likely, but are still elusive to researchers in the field.

The automatic data collection led to a dense dataset of 161 compounds with annotations for both targets, and a sparse dataset of 2191 compounds with annotation in at least one target. Applying distinct multi-label learning methods allowed to extract crucial characteristics of the compounds that are selective inhibitors of any of the two targets and to build models with good predictivity. The multi-label methods (except for binary relevance) take into account the potential interactions between labels, and therefore could bring an edge when entire protein families are of interest. To the best of our knowledge, this work pioneers the use of classifiers chain for multi-label classification in polypharmacology prediction, and might pave the way for studies across a larger set of targets.

## Methods

### Raw data

For human P-gp [UniProt: P08183], data about compounds and their inhibitory bioactivities was retrieved from the Open PHACTS Discovery Platform merely. Human BCRP [UniProt: Q9UNQ0] inhibition data was composed of data from the Open PHACTS Discovery Platform [3, 22] and of manually annotated data collected from literature sources [15], hereafter called pre-curated data. In more detail, data for BCRP was extracted from ChEMBL [1], PubChem [2], and manual search through MEDLINE. Thresholds to annotate inhibitors and non-inhibitors were applied in an assay-by-assay fashion, trying to minimize incoherence between multiple measurements. After careful cleaning, the manually curated BCRP dataset contained 433 inhibitors and 545 non-inhibitors.

### Workflow for data collection and data merging

A KNIME workflow [14] was created in order to collect data from the various sources, preprocess the data, merge it via InChIKeys [23], and filter it for the generation of the datasets of different sizes to be used by the various multi-label learning techniques. The whole workflow is available from myExperiment (http://www.myexperiment.org/workflows/4754.html), and can be flexibly adapted to any other pre-curated dataset or list of protein targets where there is data available in the open domain. The workflow queries across open data sources by means of the Open PHACTS Discovery Platform, and consists of the following steps:

#### Retrieving pharmacology data from the open domain and endpoint filtering

The “Target Pharmacology: List” API call was used to retrieve pharmacology data from ChEMBL for the protein targets under study by including a filter for the “activity_types” (activity endpoints) “IC50”, “EC50”, and “Ki”, as well as for the “activity_unit” “nanomolar”. Upstream, input was given by providing the Uniform Resource Identifier (URIs) for the UniProt identifiers of P-gp and BCRP in the form of a table. The pharmacology output was then further processed to exclude records with unspecified compound activity, and with activity values greater than 10^8^ (to avoid potential data errors). Further, activity values (for IC_50_, EC_50_, and Ki endpoints) were transformed into their negative logarithmic molar values (“-logActivity values [molar]”). The same activity endpoints are available as “pCHEMBL values” from the ChEMBL database, but in addition we also kept values with a relation different from “=”. For a binary representation (active: 1, inactive: 0), a cutoff value of “-logActivity values [molar]” of greater than five (<10 µM) was applied to define active molecules. This cutoff guarantees that activity values with a relation sign and activity >10 µM are not considered as actives. As for some data points from the Open PHACTS Discovery Platform a structural representation was missing, the “ChEMBLdb Connector Input” node was used in order to retrieve InChIKeys for all ChEMBL compound IDs in the “open” data set.

#### Importing and preprocessing pre-curated data

An sd file served as input for the import of pre-curated BCRP data. By the “String Replacer” node the terminology can be adapted to the one of the open data set (in our case “INACTIVE” was replaced by “0” and “INHIBITOR” by “1” for labeling the activity of a compound). Also column names might be adapted using the “Column Rename”. The structural input from the column “SDF Molecule” was transformed into the RDKit format, and subsequently into the InChI and InChIKey format to be able to perform the subsequent dataset merging.

#### Merging Open Data with pre-curated data

The various sources of data were merged by applying the “Concatenate” node in KNIME. By using the “Column Merger”, different columns can be united from the various input data sets (e.g. those for InChIs or activity labels).

#### Creating overlap representations of pharmacology data and heat map representations

A pivot table was generated to display bioactivities of compounds against the two targets using the “Pivoting” node in KNIME grouping rows by InChIKeys and columns by “Target name”. If several activity values are given for the same compound-target pair, only one representation (row) will be kept, preserving the mean and median activity labels as well as the list of all labels assigned to one compound-target pair. In our case of binary representation, “1” (active) and “0” (inactive) were chosen according to the label given by the pre-curated data (if available), in order to treat those annotations preferentially. In other cases, the median value of activity labels from different measurements (different ChEMBL assay IDs) was taken as the final label. In cases of 0.5 median activity labels (i.e. the compounds was attributed the two possible labels with the same frequency), compounds were removed from the dataset by applying the “Row Filter” node. The resulting heat maps were visualized with the “HeatMap (JFreeChart)” node in KNIME.

### Datasets

The dataset retrieved by the workflow can either contain no missing value (“dense dataset”, only compounds having pharmacological annotation for both transporters were kept), or can contain missing annotation for at most one of the transporters (“sparse dataset”). The “dense” dataset contains 161 compounds. The “sparse” dataset contains 2191 compounds, including the 161 compounds of the dense dataset.

The raw sparse and dense datasets retrieved by the workflow were processed using the following protocol: salts were stripped and compounds neutralized using the Wash module of MOE 2013 [24]. Mixtures, organo-metallic and compounds containing rare elements were removed. Both datasets are available for download as Additional files 2 and 3 in their washed form.

### Descriptors

All MOE 2D physicochemical descriptors were computed for the molecules of the dense dataset. In a second step, a subset of 71 interpretable descriptors was selected from the original set, in order to facilitate interpretation of the results. The exact list of the 71 selected descriptors is available in the Additional file 1. These descriptors were not selected based on information content or correlation with the dependent variable, but rather on their interpretability. Additionally, we computed the MACCS keys and the Morgan fingerprints (circular fingerprints similar to extended-connectivity fingerprints, hereafter named “ECFP”) with a diameter of 8 and folded to 1024 bits using RDKit [25].

### Multi-class classification on the dense dataset

#### Class encoding

Initially, the problem at hand can be considered a multi-label classification, with two labels: P-gp and BCRP. A compound that inhibits both P-gp and BCRP would therefore contain the two labels. This kind of machine learning question can be transformed into a more tractable problem [12]. The method we are using here is the label-powerset. Simply, each possible label combination is transformed into a class, thereby transforming the multi-label problem into a multi-class classification problem.

The dense P-gp/BCRP dataset was therefore encoded into a 4-class classification dataset. The first class (hereafter class 0) corresponds to compounds that are non-inhibitors of both P-gp and BCRP. The second class (hereafter class 1) corresponds to compounds that inhibit P-gp but not BCRP. The third class (hereafter class 2) corresponds to compounds that inhibit BCRP but not P-gp. The fourth class (hereafter class 3) corresponds to compounds that inhibit both P-gp and BCRP. Note that such problem transformation cannot be done with missing values in the labels.

#### Model building and validation

Models for predicting the four classes at the same time were built using Morgan fingerprints, MACCS keys or MOE 2D physicochemical descriptors and various algorithms (see Results). Models for distinguishing the three kinds of inhibition (classes 1, 2 and 3) were built on the small set of MOE 2D descriptors. When attribute selection was used, it was done in an embedded way inside the classifier itself to avoid over-fitting and keep the cross-validation pristine. Models for distinguishing the selective inhibitors of the two transporters (classes 1 and 2) were built on the small set of MOE 2D descriptors.

Validation was done by ten-fold cross-validation exclusively because of the reduced size of the dataset, but for the best models it was repeated 10 times with different random seeds to average results. The accuracy, Cohen’s kappa and AUC under the ROC curve are reported as measures of the quality of the classifiers.

#### Feature importance in bagging of trees

The selected model for 3-class classification was bagging of trees. In this method, many trees are built on different samples of the training set. For each tree and each feature, the percentage of data passing by the nodes in which the feature is used to split was collected. For example, a feature used at the top of the tree will see 100 % of the data. These fractions of samples they contribute to are then averaged over all trees and used to rank the features.

### Classifiers chain versus binary relevance on the sparse dataset

#### Classifiers chain

The concept of classifiers chain for multi-label learning was presented in [13]. Briefly, the list of labels (here, BCRP inhibition and P-gp inhibition) is shuffled and a model is trained using the first label and all the data for which there is an annotation for that label. The model is then used to predict this label (as a score between 0 and 1) for all compounds of the dataset (even those for which there was no information for that label). This prediction is appended to the features matrix and serves as additional descriptor for training the next model, on the second label. This in turn can be used to predict the next label, etc. until all labels in the shuffled list have been learned.

#### Binary relevance

Binary relevance corresponds to the baseline of multi-label classification. It assumes independence of labels, and therefore building independent models for each label and using them together for the final prediction is a simple and efficient approach [26].

#### Model building and evaluation

In the present implementation, the same modeling algorithms were used for both labels. We used logistic regression with default parameters, random forest with 100 trees, and a support vector machine with polynomial kernel of degree two from scikit-learn [27] as base classifiers. The features used were the Morgan fingerprints as described in the “Descriptors” section. Accuracy, MCC and AUC were computed using the macro-averaging procedure [11] after running ten-fold cross-validation. In macro-averaging, the number of true positive, true negative, false positive and false negative are computed label by label, then accuracy, MCC and AUC are computed, also label by label. These values are then averaged. Note that this evaluation cannot be compared with the direct evaluation of the label-powerset models, since there the accuracy represents all exact matches between the predictions and the real class. It is stricter than the macro-averaged measures. This method cannot be applied here since there are missing labels in the training set. The same evaluation method is applied to both classifiers chain and binary relevance (each individual model is evaluated by the same cross-validation folds and then the individual measures are averaged).

The python code to build and validate such classifiers as well as instructions for running it is available as Additional files 1 and 4.

### Scaffolds analysis

Bemis–Murcko scaffolds [16] were extracted with RDKit KNIME node “RDKit Find Murcko Scaffolds” from both the dense and sparse datasets. Compounds sharing the same scaffold were grouped together and their pharmacological annotations were retrieved. Heat map representations for the most prominent scaffolds were visualized with the “HeatMap (JFreeChart)” node in KNIME.

### Software

The data collection workflow was implemented in KNIME version 2.11 [14]. The Open PHACTS API version 1.5 [28] was used to query across integrated public data sources: ChEMBL [1], ChEBI [29], DrugBank [30], Chemspider [31], Gene Ontology [32], WikiPathways [33], Uniprot [34], ENZYME [35], and ConceptWiki [36]. OPS-KNIME nodes (version 1.1.0) are available from [37]. Data cleaning and descriptor calculations were performed using MOE 2013 [24] and RDKit 2014.09.2 [25]. Multi-class classification models were built using the Weka 3.7.7 package [38] and chemical space projections were done with CheS-Mapper 1.9.0 [18]. Classifiers chain and binary relevance models were built using an in-house script using the scikit-learn python library [27] version 0.16.1. The distribution plots of the descriptors were done using an in-house python script and the matplotlib library [39] version 1.14.3. The Bemis-Murcko scaffold analysis was done in LibreOffice Calc 4.2.8.2 and the structures were drawn in Marvin 5.11.3, 2012, ChemAxon (http://www.chemaxon.com).

## Additional files

The following additional data are available with the online version of this paper:

10.1186/s13321-016-0121-y The list of descriptor names, instructions on how to run the python script, the distribution plots for the important descriptors, the heat map of activities for the dense dataset, the structure of the over-represented scaffolds in the sparse dataset, a 2D representation of a PCA run on Morgan fingerprints (ECFP-like) for both dense and sparse datasets, and the structures of the 9 misclassified compounds.
10.1186/s13321-016-0121-y The dense dataset (161 compounds) with the label-powerset transformation of the classes.
10.1186/s13321-016-0121-y The whole dataset (2191 compounds) as used in the binary relevance—chains of classifiers script.
10.1186/s13321-016-0121-y The python script to compare binary relevance with chains of classifiers.
