# Supplementary material for: Selectivity profiling of BCRP versus P-gp inhibition: from automated collection of polypharmacology data to multi-label learning
Source: J Cheminform. 2016 Feb 4;8:7. doi: 10.1186/s13321-016-0121-y (PMC4743411; doi:10.1186/s13321-016-0121-y)

Supplementary information

71 MOE descriptors

The reduced set of 71 descriptors used in the 3-class and 2-class classification models is the following:

apol, a_acc,a_acid,a_aro,a_base, a_count, a_don,a_donacc,a_heavy, a_hyd, a_IC, a_IC, a_nB, a_nBr, a_nC, a_nCl, a_nF, a_nH, a_nI, a_nN, a_nO, a_nP, a_nS, balabanJ, bpol, b_1rotN, b_1rotR, b_ar, b_count, b_double, b_heavy, b_max1len, b_rotN, b_rotR, b_single, b_triple, chiral, density, diameter, FCharge, lip_violation, logP(o/w), logS, mr, mutagenic, nmol, opr_brigid, opr_leadlike, opr_violation, petitjean, petitjeanSC, radius, reactive, rings, rsynth, SlogP, SMR, TPSA, vdw_area, vdw_vol, vsa_acc, vsa_acid, vsa_base, vsa_don, vsa_hyd, vsa_other, vsa_pol, Weight, weinerPath, weinerPol, Zagreb

How to install and run the python script

The script was written in python 2.7.10 but probably runs on any python 2.6 and plus. Not on python 2.3.

Dependencies:

The script requires:

- a recent **RDKit** version
- a recent **scikit-learn** version
- the **pandas** library
- **numpy**

The kind of training set you can feed it:

Exclusively an sd file with all the compounds to train on, with one identification property (like a molecular index or a unique name). The rest of the properties are the binary labels to learn, encoded as 0 for inactive, 1 for active and -1 for missing. No other property should be present in the file.

Where you indicate the path to your sd file(s)

At the beginning of the script, after the imports, you will see the following:

```
##########################   PATHS to the dataset: TO CUSTOMIZE ###############################
ALL_DATA = '/home/floriane/Projects/thesis-flo/experiments/Pgp-BCRP-BSEP_profiling/CHEMBL20_data_2/sparse_dataset.sdf'
################################################################################################
```

This is where you set up the path to your own file.

How to run the script

Once all dependencies are installed and the path to the sd file is properly set, go to the directory where the script is stored and in a terminal write:

>>python chain_of_classifiers.py

This will automatically run the script that reproduces the results shown in the paper (if the "ALL_DATA" variable points to the proper file).

Supplementary Figures

Figure SI-1: Binary heat map representation of compounds of the dense dataset (2191 compounds) measured for their inhibitory activity against BCRP and P-gp (red, inhibitor; blue, non-inhibitor; abscissa, targets; ordinate, compounds with exemplary ChEMBL IDs):

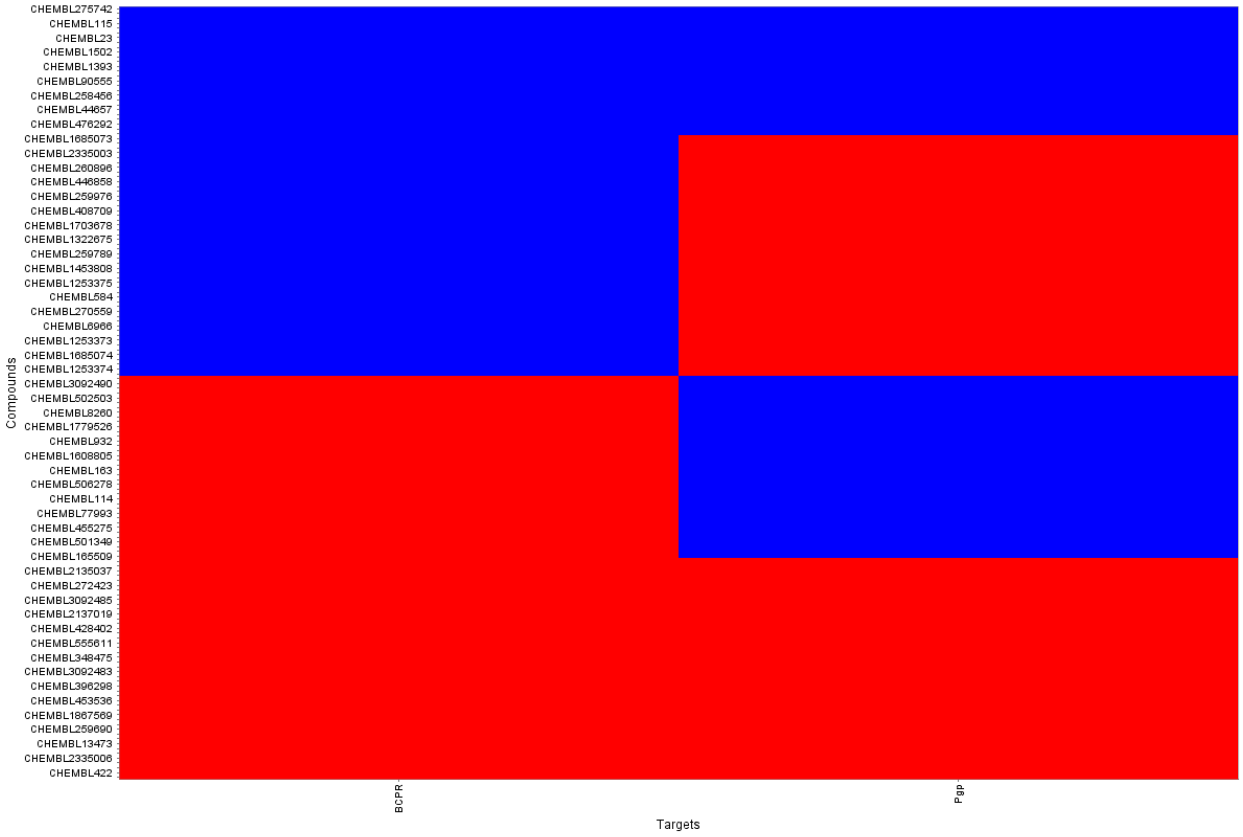

Figure SI-2: Structure and class presence of the 13 scaffolds with more than 20 representatives in the sparse dataset.

In green, the proportion of compounds annotated with P-gp inhibition. In yellow, the proportion of compounds annotated with P-gp inactivity. In orange, the proportion of compounds annotated with BCRP inhibition. In blue, the proportion of compounds annotated with BCRP inactivity.

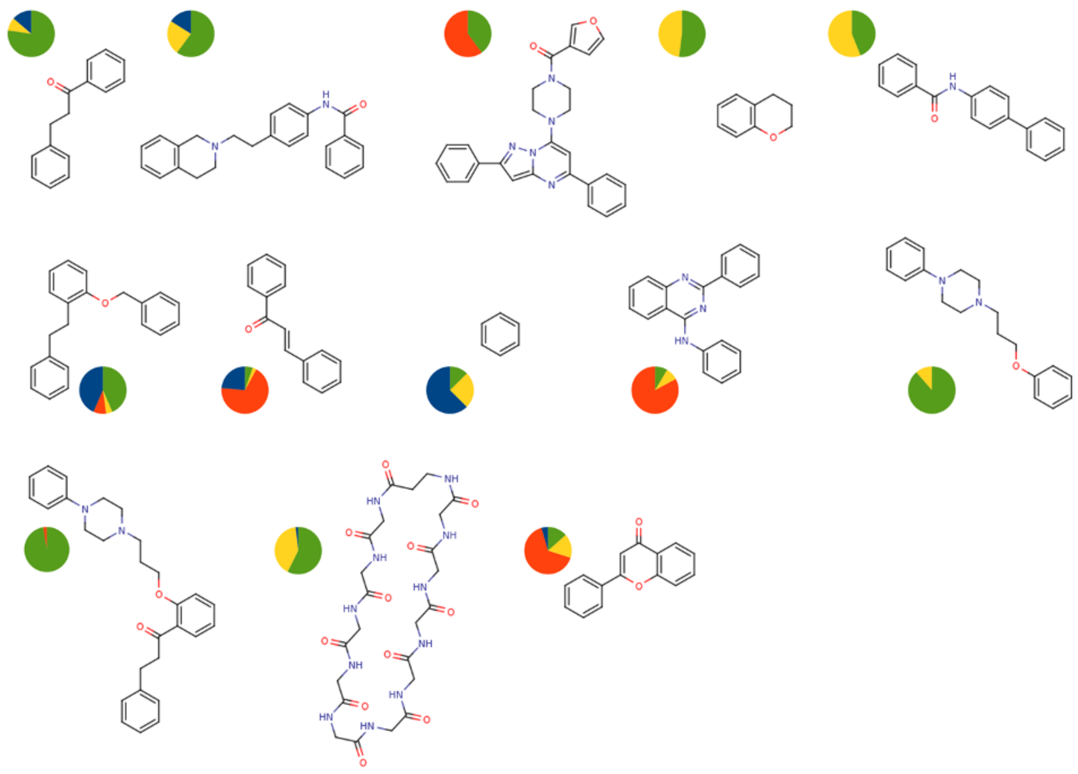

Figure SI-3: Projection of the dense dataset (yellow dots) over the PCA transformations obtained for the sparse dataset (black dots) using ECFP-like fingerprints

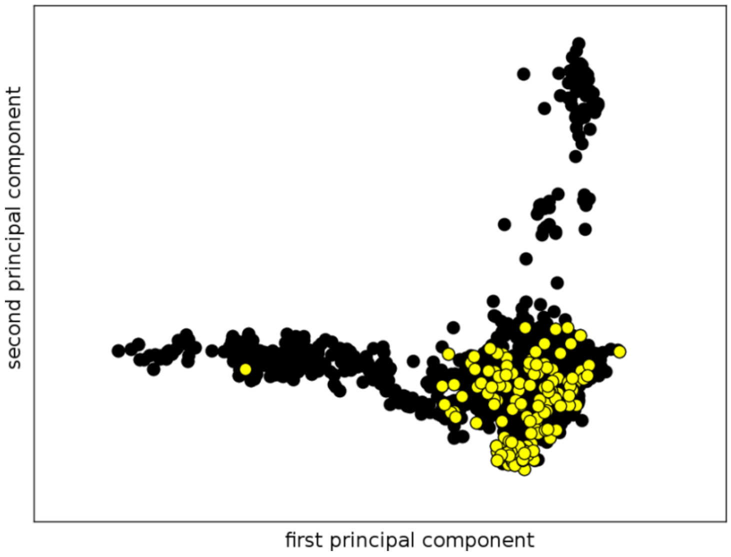

Distribution plots

- **Figure SI-4**: Distribution of a_hyd among classes 1, 2 and 3

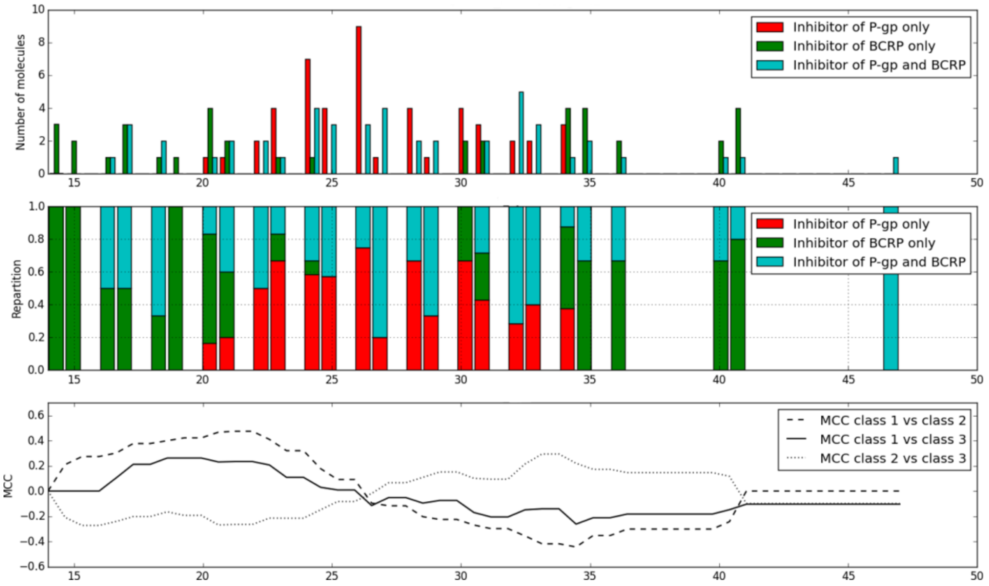

- **Figure SI-5**: Distribution of a_donacc among classes 1, 2 and 3

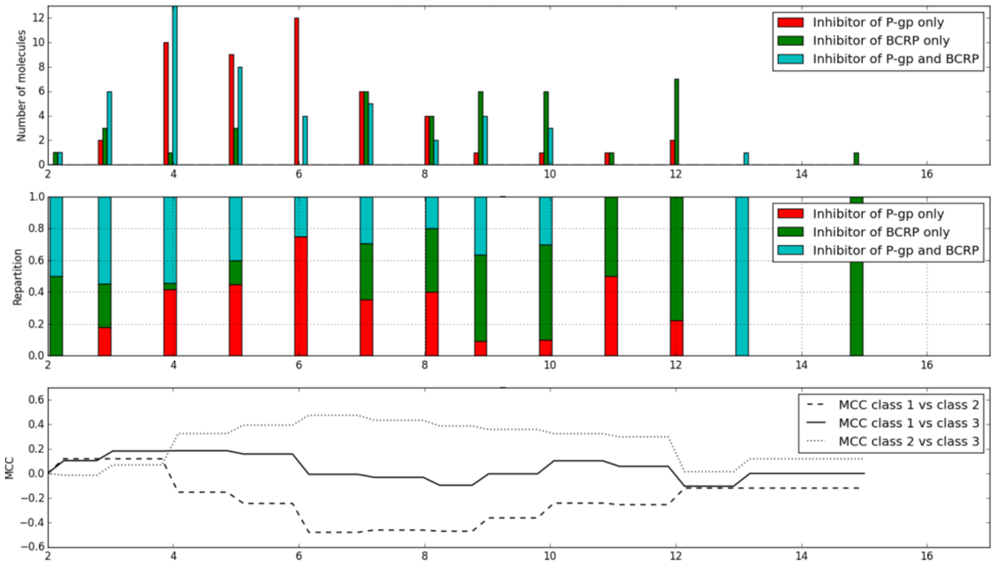

- **Figure SI-6**: Distribution of weinerPath among classes 1, 2 and 3

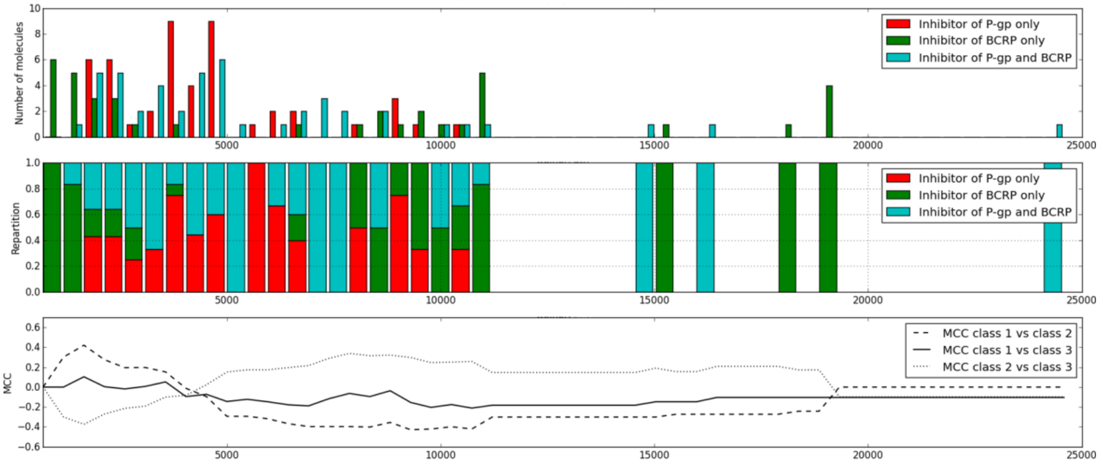

- **Figure SI-7**: Distribution of a_aro among classes 1, 2 and 3

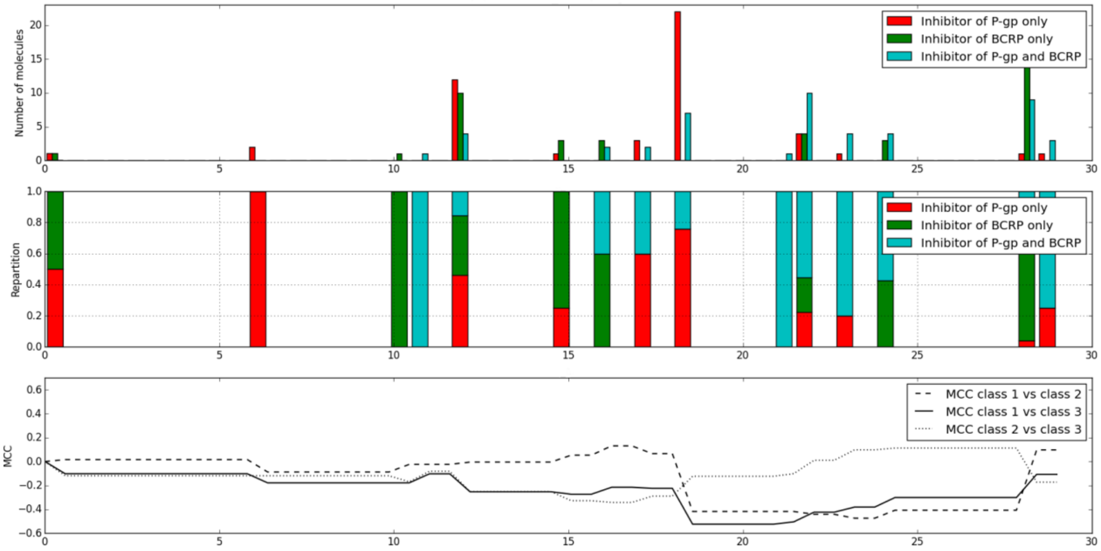

Figure SI-8: Distribution of balabanJ among classes 1, 2 and 3

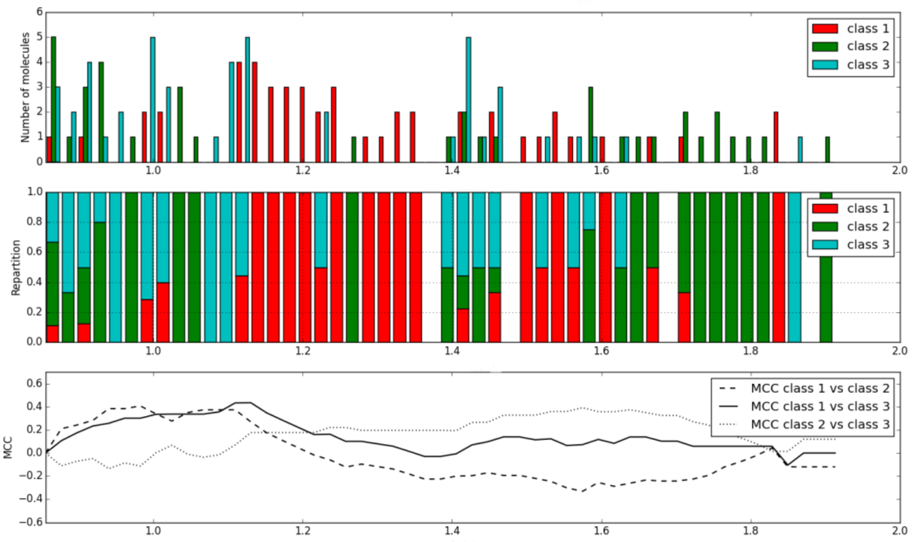

Figure SI-9: Distribution of vsa_acc among classes 1, 2 and 3

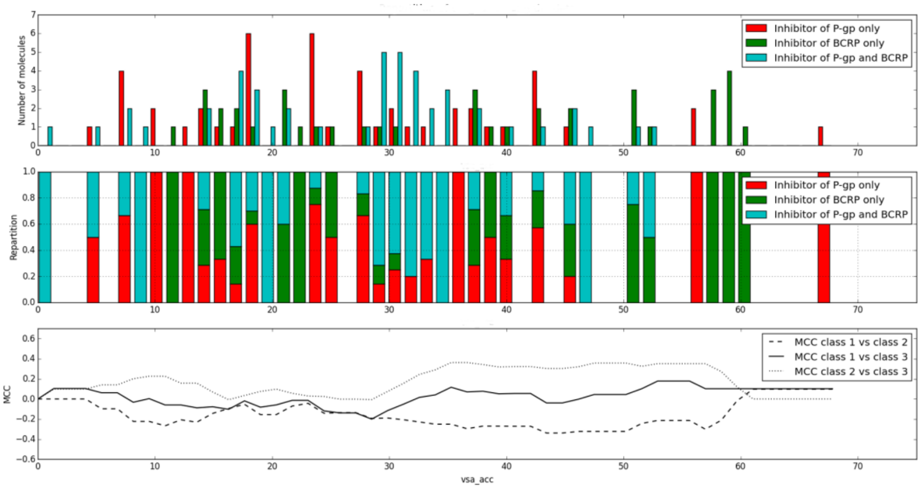

Figure SI-10: Structures of the 9 compounds misclassified by the JRip rules to separate BCRP-selective from P-gp-selective inhibitors. We indicate the CSID (unique identifier), the class, the number of aromatic atoms and the number of hydrophobic atoms besides each structure.

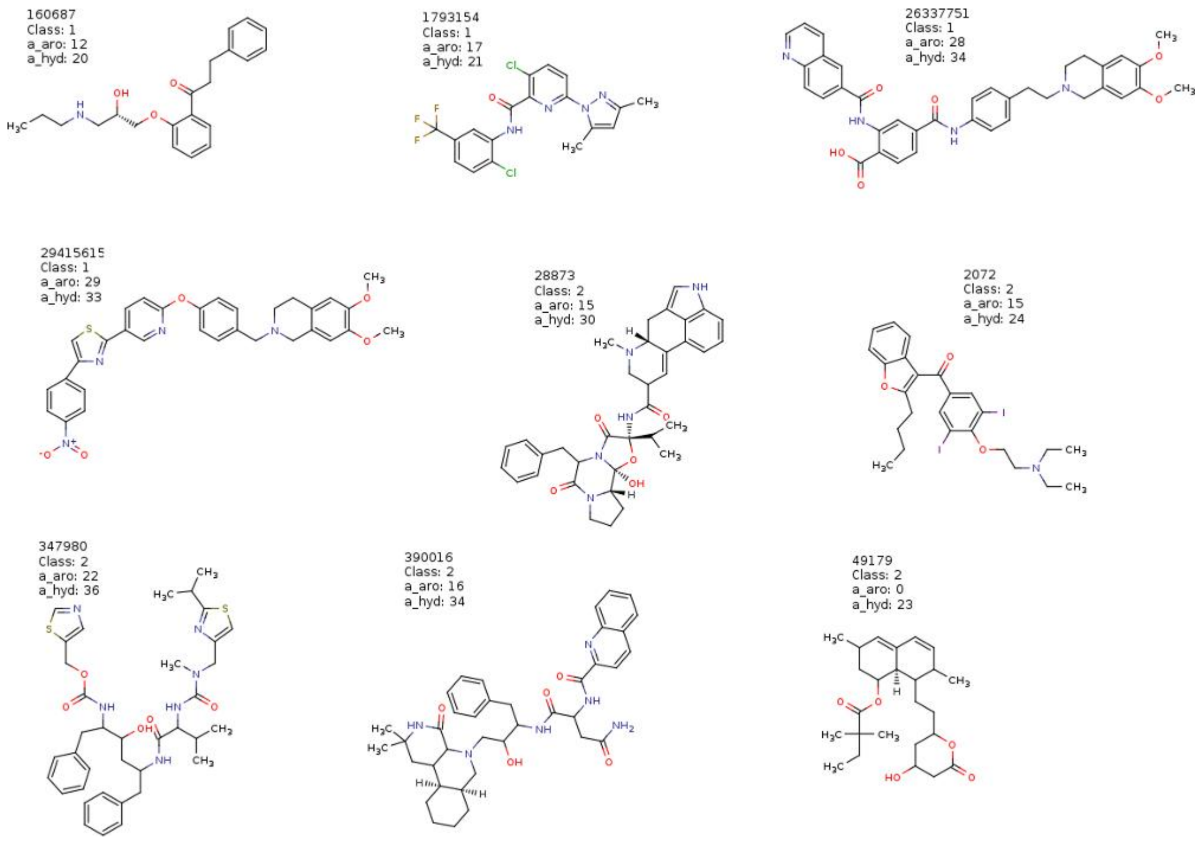

Figure SI-11: Number of hydrophobic atoms plotted as a function of the number of aromatic atoms for compounds in classes 1 and 2 (selective inhibitors of P-gp or BCRP); the lines show the decision boundaries of the JRip model. In red, compounds in class 1; in green, compounds in class 2.

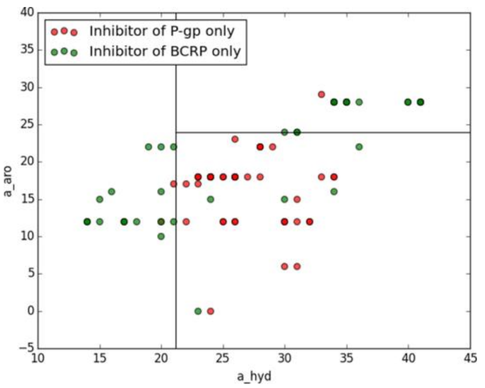

Supplement: Supplementary file 1 — 10.1186/s13321-016-0121-y The list of descriptor names, instructions on how to run the python script, the distribution plots for the important descriptors, the heat map of activities for the dense dataset, the structure of the over-represented scaffolds in the sparse dataset, a 2D representation of a PCA run on Morgan fingerprints (ECFP-like) for both dense and sparse datasets, and the structures of the 9 misclassified compounds. [file 13321_2016_121_MOESM1_ESM.pdf]
